# Anticoagulation Drug Therapy: A Review

**DOI:** 10.5811/westjem.2014.12.22933

**Published:** 2015-01-12

**Authors:** Katherine Harter, Michael Levine, Sean O. Henderson

**Affiliations:** University of Southern California, LA+USC Medical Center, Department of Emergency Medicine, Los Angeles, California

## Abstract

Historically, most patients who required parenteral anticoagulation received heparin, whereas those patients requiring oral anticoagulation received warfarin. Due to the narrow therapeutic index and need for frequent laboratory monitoring associated with warfarin, there has been a desire to develop newer, more effective anticoagulants. Consequently, in recent years many novel anticoagulants have been developed.

The emergency physician may institute anticoagulation therapy in the short term (e.g. heparin) for a patient being admitted, or may start a novel anticoagulation for a patient being discharged. Similarly, a patient on a novel anticoagulant may present to the emergency department due to a hemorrhagic complication. Consequently, the emergency physician should be familiar with the newer and older anticoagulants. This review emphasizes the indication, mechanism of action, adverse effects, and potential reversal strategies for various anticoagulants that the emergency physician will likely encounter.

## INTRODUCTION

**Figure 1:**
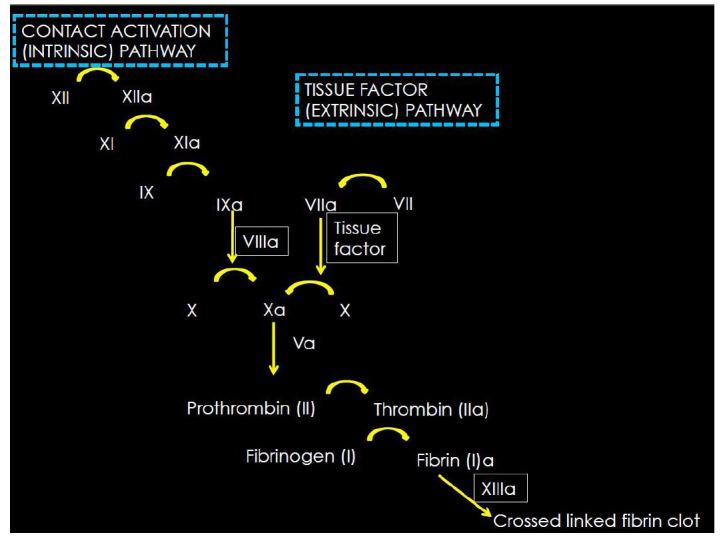
The coagulation cascade.

During routine homeostatic conditions, the human body maintains a constant balance between thrombus formation and destruction. This equilibrium is maintained by a complex interaction between platelets and the vascular endothelium, the coagulation cascade, and the fibrinolytic system. The coagulation cascade (Figure 1) involves an interaction between the contact activation pathway (previously called the intrinsic system), and the tissue factor pathway (previously the extrinsic system). These two seemingly independent pathways lead to the conversion of factor X to Xa, which is the start of the common pathway. This common pathway converts prothrombin to thrombin, which subsequently catalyzes the formation of fibrin and ultimately leads to the stabilization of aggregated platelets to form a stable clot.^1^,^2^

**Figure 2:**
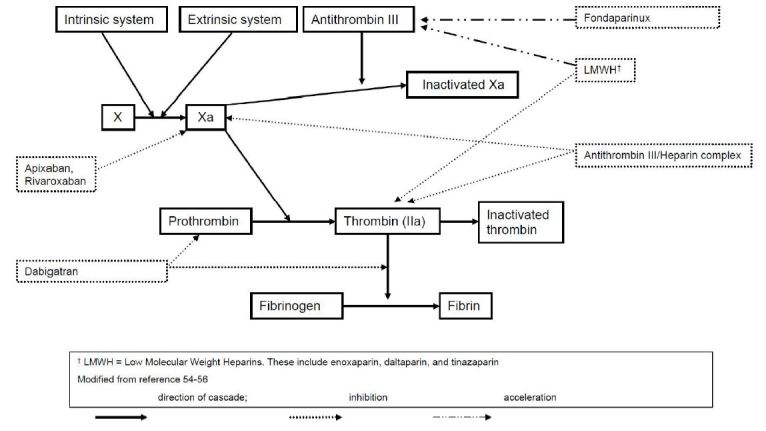
Site of action of drugs. Modified, with permission, Gresham C, Levine M, Ruha AM.^17^

Historically, vitamin K antagonists, such as warfarin, were the only anticoagulants widely available for human use. It has been estimated that more than 65,000 patients are treated in U.S. emergency departments (ED) annually for warfarin-related hemorrhage.^3^ Because of this high rate of bleeding, along with the drug’s narrow therapeutic index and the need for frequent monitoring, there has been a desire to create safer anticoagulants without such strict drug monitoring. Consequently, there have been several novel anticoagulants (NACs) developed, including direct thrombin inhibitors (e.g. dabigatran), and factor Xa inhibitors (e.g. rivaroxaban, apixaban), designed to target different points of the coagulation cascade (Figure 2).^4^,^5^

**Figure 3:**
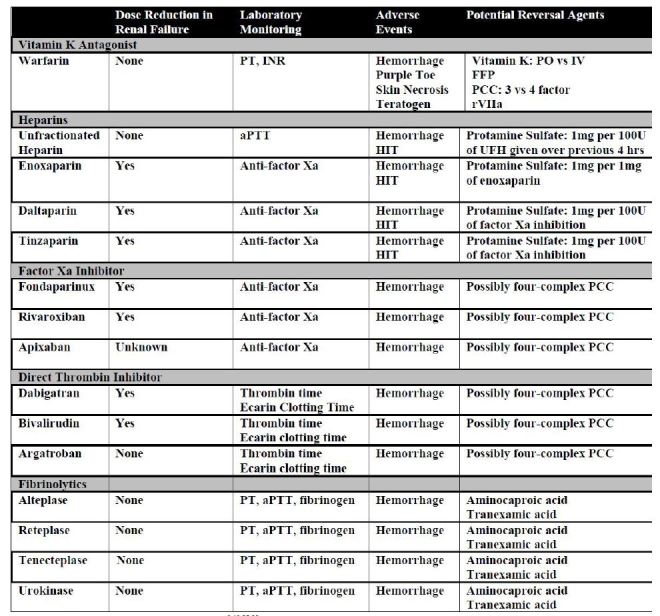
Comparison table for anticoagulants.^9^,^19^,^25^,^38^ *PT*, pro-thrombin time; *INR*, international normalized ratio; *HIT,* heparin-induced thrombocytopenia; *PO*, oral administration; *IV*, intravenous; *FFP,* fresh frozen plasma; *aPTT,* activated partial thromboplastin time; *UFH*, unfractionated heparin; *PCC*, prothrombin complex concentrates

As NACs become more pervasive in the clinical setting, used for both therapeutic and prophylactic purposes, it will become essential for the emergency physician to become aware of the indications to start specific drugs, as well as unique complications and recommended reversal methods for such agents. An intimate knowledge of these drugs will be required for the ideal management. Unfortunately, while the clinical efficacy of NACs has been established, much less is known about the risks of adverse reactions as well as the ability to reverse these agents.^6^ Figure 3 below summarizes the most widely-used anticoagulants; they will be discussed in this article. This article provides a review of the literature as it focuses on both the risks associated with anticoagulants, as well as reversal agents of the most commonly used NACs to help guide management in the emergency setting.

### Vitamin K antagonists

Vitamin K antagonists (VKAs) such as warfarin function by blocking the vitamin K-epoxide reductase, thereby preventing formation of the active form of the vitamin K-dependent clotting factors.^7^ The VKAs have an initial pro-thrombotic effect, by initially blocking proteins C and S, followed by a delayed antithrombotic effect, through the inhibition of coagulation factors II, VII, IX, and X.^7^

#### Warfarin

Federal Drug Administration indications for use include long-term anticoagulation following a thrombotic event or prevention of thrombotic events in patients at high risk, including post-operative states, atrial fibrillation, and those with artificial valves.^8^ Because of the initial pro-coagulant effect, if rapid anticoagulation is required, warfarin is paired with a rapid-acting parenteral anticoagulant, which can be discontinued after therapeutic levels are achieved and stable over the course of 24 hours.

Warfarin is taken orally, at doses typically ranging from 5–10mg daily, tailored based on the international normalized ratio (INR), the universal monitoring index based on pro-thrombin time (PT). Warfarin is primarily metabolized through the P450 system.^9^ Induction or inhibition of the isoenzymes involved with warfarin’s metabolism can potentially increase the INR significantly.^7^ Furthermore, alterations in oral vitamin K consumption can create significant fluctuations in the INR.^10^

##### Side Effects and Reversal Agents

Hemorrhage is the most significant adverse effect associated with warfarin and is directly related to the level of INR; the risk of hemorrhage is increased if the INR is greater than five.^7^ Risk factors for warfarin-related hemorrhage include advanced age, serious co-morbid conditions including cancer, chronic kidney disease (CKD), liver dysfunction, arterial hypertension, prior stroke, alcohol abuse, and the concomitant use of antiplatelet or other drugs.^7^ In the event of hemorrhage, the anticoagulant effects of warfarin can be reversed with the administration of vitamin K (phytonadione), fresh frozen plasma (FFP) or prothrombin complex concentrates (PCCs).^12^,^13^ In addition, recombinant factor VIIa (rfVIIa) has been suggested as a possible reversal agent. While the use of rfVIIa has been demonstrated to provide a rapid reduction in the INR, its use is not associated with improved clinical outcomes.^14^,^15^

### Heparins

Antithrombin III (AT3) is a peptide that inhibits several of the activated clotting factors. Drugs that augment the function of AT3 serve as anticoagulants. Unfractionated heparin (UFH) binds to and increases the activity of antithrombin III by inducing a conformational change to Factor Xa, which ultimately leads to inhibition at Xa and IIa in a 1:1 ratio.^16^ Unfractionated heparin also has some inhibition on factors IXa, XIa, XIIa.^17^ Low molecular weight heparins (LMWH), which also bind AT3, are smaller and have a higher proportional impact on Xa, versus IIa, in a 3:1 or 2:1 ratio.^16^,^17^ As a result of this inhibition, both the UFH and LMWH ultimately inhibit thrombin activation.

#### Unfractionated Heparin (UFH)

UFH is indicated for numerous conditions including the treatment and prophylaxis of venous thromboembolisms (VTE), thrombus prophylaxis in atrial fibrillation, and treatment of disseminated intravascular coagulation.^18^ Unlike warfarin, UFH is administered parenterally, both subcutaneous for its prophylaxis use and as a continuous intravenous infusion when used therapeutically. UFH has much faster onset of action as compared to warfarin; when used intravenously, therapeutic efficacy occurs almost immediately, while therapeutic efficacy is reached within 20–60 minutes when administered subcutaneously.^9^ UFH has a shorter half- life than warfarin, and does not require dosage adjustment in renal failure.^9^

##### Side Effects and Reversal Agents

Hemorrhage is a main adverse event in those receiving UFH. The incidence of major bleeding varies based on the indication of its use, dosage and route of administration. However, on average, UFH is associated with a 2.0% incidence of major bleeding when used therapeutically for VTE.^19^ While major bleeding can be potentially fatal, UFH can be reversed with the administration of protamine sulfate. Typically, protamine is dosed based on the amount of UFH administered, not based on laboratory abnormalities. A dose of 1mg will reverse 100 units of UFH.

Another significant and well-documented adverse outcome of UFH use is the development of heparin-induced thrombocytopenia (HIT). A detailed discussion of HIT, however, is beyond the scope of this review. Nonetheless, treatment options for HIT include discontinuation of UFH, and the subsequent use of a different class of NAC, either a direct thrombin inhibitor (e.g. argatroban) or a factor Xa inhibitor (e.g. fondaparinux).

#### Low Molecular Weight Heparin (LMWH)

The LMWH are parenterally-administered drugs, and include dalteparin, enoxaparin, and tinzaparin. Compared with UFH, the LMWH have the advantage of a more predictable dose-response curve.^17^ Consequently, the LMWHs are administered at a fixed dose, based on total body weight, and do not require tight regulation and monitoring as is indicated with warfarin and UFH.^17^ These drugs have near 100% bioavailability and reach peak levels 2–4 hours after subcutaneous administration.^9^,^17^ They have a half-life of 3–4 hours and are eliminated primarily (80%) via renal clearance, thus necessitating dose reduction considerations in patients with renal insufficiency.^9^ Additionally, since dosing is based on total body weight, rather than ideal body weight, dosing complications arise in obese patients.^17^ While therapeutic monitoring is not routinely indicated, in cases of renal insufficiency, obesity, or when iatrogenic overdose is a concern, antifactor Xa levels can be used to monitor LMWH.^9^,^17^ Ideally, the antifactor Xa level should be obtained four hours after the administration of the LMWH.

##### Side Effects and Reversal Agents

Acute bleed is the major risk associated with LMWH. When used prophylactically the incidence of major bleeding associated with the LMWH is approximately 1.5–1.7%.^19^,^20^ The incidence of major bleeding associated with therapeutic dosage of the LMWH is slightly higher at approximately 2%, with even higher incidences observed when used to treat acute coronary syndrome (ACS).^19^ In the event of a major bleed, protamine sulfate can be used as a partial reversal agent and can reverse at most 60% of the anticoagulation effect of LMWH.^19^ Initial doses of 1mg per 100 units of antifactor Xa should be administered within eight hours of LMWH administration. A second dose of 0.5mg per 100 units antifactor Xa can be repeated.^17^ For significant bleeding associated with LMWH, cryoprecipitate and fresh frozen plasma is also recommended.^17^,^19^

### Factor Xa inhibitors

Factor Xa inhibitors are used for prophylaxis and treatment of VTE, as well as for prophylaxis of embolic disease in non-valvular atrial fibrillation, and as an alternative anticoagulant in the setting of HIT. These drugs inhibit factor Xa, the first step in the common pathway, either directly or indirectly. The inhibition occurs in a dose-dependent manner.^21^ Apixaban and rivaroxiban, directly bind to the active site of factor Xa, thereby inhibiting both free and clot-associated factor Xa. These drugs also inhibit prothrombinase activity.^5^ Indirect Xa inhibitors, such as fondaparinux, bind to AT3, resulting in a conformational change, thereby inhibiting factor Xa without having any effect on IIa.^17^ Fondaparinux is primarily eliminated unchanged in the urine. Thus, its use in patients with renal insufficiency is contraindicated as its use in this patient population may increase the risk of hemorrhage.

There are no specific laboratory parameters available to monitor the anticoagulant impact of factor Xa inhibitors. A dose-dependent prolongation of aPTT and PT may be seen 1–4 hours after administration of direct Xa inhibitors such as rivaroxiban, matching the peak plasma level; however, this increase is short lived and in general PT, aPTT and bleeding time should not be affected at therapeutic levels of these drugs.^9^ Supratherapeutic concentrations of Xa inhibitors, however, have been associated with a dose-dependent increase in PT.^9^ This increase in PT does not directly correlate with the increase in PT secondary to VKAs, and there is not a consistent conversion between the PT and the INR with these drugs.^22^ Antifactor Xa levels were originally designed and calibrated for LMWH; however, they can also be used to monitor or confirm overdose of factor Xa inhibitors.^9^ This test must be specifically calibrated for Factor Xa inhibitors, as the results of the antifactor Xa level is assay specific.^17^,^23^

#### Side Effects and Reversal Agents

Adverse events related to Xa inhibitors include hemorrhage, as is the case with all anticoagulants. Thrombocytopenia has also been reported following the use of Xa inhibitors; however, the mechanism is unclear.^17^ While no specific reversal agent exists, both rVIIa and PCC have been proposed.^9^,^19^ The Thrombosis and Hemostasis Society of North America suggests that four-factor PCC may be the best option currently available.^24^ The German Society of Neurology recommends PCC for reversal of factor Xa inhibitor-induced coagulopathy. However, at present, there is insufficient data to clearly support any reversal agent or to develop a standard of care.^25^

### Direct thrombin inhibitors (DTIs)

As their name implies, the direct thrombin inhibitors (DTIs) inhibit the intrinsic activity of the thrombin. Unlike heparin, which also inhibits thrombin, the DTIs do not require a factor, and can inhibit thrombin directly.^7^,^26^ Most direct thrombin inhibitors are administered parenterally, including argatroban, bivalirudin; however, dabigatran is orally administered. These drugs are used for prophylaxis and treatment of VTE and ACS, and for prophylaxis of thrombus formation in non-valvular atrial fibrillation. They are also used as anticoagulation alternatives in the setting of HIT. Dabigatran, the only orally available DTI, is approved for treatment of VTE in patients treated with concomitant parenteral anticoagulation for at least five days, and for the treatment of thrombus secondary to non-valvular atrial fibrillation.

Laboratory evaluation of the DTIs includes measurement of a thrombin time (TT) or ecarin clotting time (ECT).^29^ However, these tests are not widely available, thereby limiting their applicability, particularly in the emergency setting. The Hemoclot test is a diluted thrombin time assay designed specifically as an assay for the DTIs; however, like the TT and ECT, this test is not routinely available.^30^,^31^ In the clinical setting, activated partial thromboplastin time (aPTT) can be used as a surrogate to monitor the effect of the DTIs; aPTT increases following a non-linear dose response curve and plateaus at higher concentrations of DTIs. Thus, a normal aPTT excludes the presence of significant amounts of a DTI, but the degree of elevation of the aPTT does not necessarily correlate with the degree of DTI-induced coagulopathy.^29^

#### Side Effects and Reversal Agents

The primary toxicity of patients on DTIs is hemorrhage, including gastrointestinal bleeding and intracranial hemorrhage. The rate of bleeding is dose dependent, and is more common in those over 75 years of age.^27^,^28^ Like many other NACs, no specific antidotes exist. The American College of Cardiology Foundation and the American Heart Association recommend transfusion of packed red blood cells and FFP, in addition to surgical intervention, if feasible, to control bleeding.^32^ However, given that FFP contains factor II, which is inhibited from activation by DTIs, the use of FFP is unlikely to be beneficial.^25^ For patients with impaired renal function who have life-threatening bleeding following dabigatran-induced coagulopathy, hemodialysis has been recommended by some experts.^29^ Others have suggested that in the event of significant bleeding, the use of a four-complex PCC may be the most effective option; however, there is limited evidence-based data.^25^

### Fibrinolytics

The antithrombotic effect of fibrinolytics, which include tissue plasminogen activator (tPA) and urokinase, is achieved by inducing the conversion of inactive plasminogen into the active enzyme plasmin, which degrades the fibrin matrix responsible for stabilizing a thrombus.^33^ Recombinant forms of tPA and urokinase have been manufactured as fibrinolytics. Alteplase, an unmodified form of human tPA, along with reteplase and tenecteplase, a modified form of human tPA, are the most commonly used drugs in this class.^34^ Common uses of these drugs include the treatment of acute cerebrovascular accidents (CVA), myocardial infarction, pulmonary emboli, as well as to dissolve thrombi in indwelling catheters. Following administration of fibrinolytics, an increase in the PT/INR and aPTT can be observed, along with a corresponding decrease in the fibrinogen; however, there are no specific laboratory indices to precisely measure the anticoagulant effect of fibrinolytics.

#### Side Effects and Reversal Agents

The incidence of hemorrhage varies depending on the indication for the fibrinolytic. When used for acute CVA, tPA is associated with symptomatic intracranial hemorrhage at a rate of approximately 6%.^35^,^36^ However, when tPA is given to those with healthy brains, the rate of such hemorrhage is much lower.^37^

In the event of acute hemorrhage the administration of blood products, including FFP, PCC, and platelets, have been found to have poor efficacy, and other agents, including tranexamic acid (TXA) and epsilon-aminocaproic acid (EACA), have been considered.^38^ TXA and EACA are both structurally similar to the amino acid lysine and inhibit fibrinolysis by competitively inhibiting plasminogen activation.^38^

## CONCLUSION

Acute hemorrhage is the most feared adverse event associated with all anticoagulants. While it is relatively uncommon that patients present with a life-threatening hemorrhage while on systemic anticoaguation, prompt recognition and management is vital. As the NAC become more frequently used in clinical settings, it will be imperative that the emergency physician has a thorough understanding of these agents, and is knowledgeable about potential reversal strategies, when available.
